# Editorial: Translational behavioral approaches in animal models of psychiatry

**DOI:** 10.3389/fnbeh.2023.1200691

**Published:** 2023-05-16

**Authors:** Stamatina Tzanoulinou, Johannes Passecker, Antonios Stamatakis, Anastasia Diamantopoulou

**Affiliations:** ^1^Department of Biomedical Sciences, Faculty of Biology and Medicine, University of Lausanne, Lausanne, Switzerland; ^2^Center for Chemistry and Biomedicine, Innsbruck Medical University, Innsbruck, Tyrol, Austria; ^3^Department of Nursing, School of Health Sciences, National and Kapodistrian University of Athens, Athens, Greece; ^4^Institute of Neurophysiology, Faculty of Medicine, Goethe University Frankfurt, Frankfurt am Main, Germany

**Keywords:** animal model, translation, behavior, psychiatric disorder, brain function

During the past two decades, the number of animal models of psychiatric disorders has grown exponentially, whereas at the same time investment of pharmaceutical companies on neuropsychiatric research has been stagnating. Limited success with efficacious novel treatments for neuropsychiatric disorders can be in part attributed to the unsolved challenge of translating symptom specific mechanisms into animal models (or *in-vitro* models). Nevertheless, recently approved drug treatments for psychiatry like esketamine for treatment-resistant depression and brexanolone for postpartum depression owe their approval to extensive, targeted and well-replicated research on appropriate animal models (Ramaker and Dulawa, 2017; Maguire, 2019). Therefore, despite the fact that animal models are rarely able to capture a human disorder in its entirety, they are undoubtedly useful to model causative factors (i.e., a mutation that increases substantially disease risk) and domain-specific aspects of a disorder that may manifest in an equivalent mechanistic manner in animals. However, usefulness and effectiveness of preclinical studies in guiding future drug treatments is maximized when studies using animals to model psychiatric disorders employ behavioral constructs with high cross-species transferability but also introduce an ethological relevance into a well-controlled experimental design. The following compilation of original research pieces along with reviews and perspective articles offers some invaluable insights into the above matters.

Petković and Chaudhury present a comprehensive review on the essential features of animal models of depression and mood disorders with a focus on the translational value of paradigms and behavioral tests. They present paradigms of chronic unpredictable mild stress, learned helplessness, social defeat and restraint stress, as well as, prenatal and neonatal stress as models of psychopathology. Finally, the authors emphasize the need to undertake complex brain circuit analyses in the context of behavioral approaches, while, on the other hand, research from human patients will be necessary to validate those animal models.

Rincón-Cortés and Grace give an overview of postpartum depression (PPD) models and provide a summary of evidence supporting the view of the mesolimbic dopamine system as a key node of depressive-like and anhedonic responses involved in PPD. They review genetic, stress-induced, diet-based models, as well as novel PPD paradigms, such as permanent pup removal (Rincón-Cortés and Grace, 2021) and postpartum scarcity-adversity (Rincón-Cortés and Grace, 2022). Specifically, dams in both models showed reduced number of active dopamine neurons in the ventral tegmental area, suggesting an attenuated DA system activation. Similar perturbations of the DA system have been observed in PPD in humans (Post and Leuner, 2019). This overview highlights the brain DA system as a factor contributing to motivation and reward-related deficits comorbid with PPD and emphasizes it as a target for potential treatments.

Using an elegant design of pavlovian-to-instrumental transfer (PIT), Derman and Lattal show that an acute trauma has persistent effects on general motivational processes, while uncovering sex-specific differences in a paradigm of sensory-specific PIT. This work is performed in the context of stress-enhanced fear learning (SEFL) which allows to dissociate the memory for the trauma and the effects of the stress originating from that trauma. Thus, this type of paradigms can be relevant for modeling key features of post-traumatic stress disorder (PTSD).

Haller focuses on models of aggression-related psychopathologies, arguing that there is a further need to study pathological aggression in more appropriate contexts. That is because, despite the fact that studies on naturally-occurring functional and adaptive aggressive behaviors such as defending one's territory or offspring and establishing and/or maintaining social ranks have indeed contributed to the understanding of aggression, psychopathological aggression that does not serve adaptive purposes seems to correspond to different neurobiological correlates. Hence, animal models of psychopathological aggression (Tóth et al., 2008; Walker et al., 2017), induced by environmental (i.e., etiological factors), such as exposure to traumatic experiences, can be fundamental in elucidating neural mechanisms of abnormal aggression in humans.

Adding a perspective of the use of non-human primates (NHP) as animal models for neuropsychiatric diseases, Ausderau et al., offer a critical overview of the use of marmosets and macaques, being the most commonly used NHPs for biomedical studies, on anxiety and depression research. They emphasize the importance of taking into account age-appropriate activities and responses when using tests of anxiety or depression features, as well as evaluating those under the prism of comorbidity in NHP models of disease.

Lipp and Wolfer substantiate a decades-long viewpoint on interpretation issues in behavioral translational studies. They argue that translational studies would benefit from using multiple different ethologically-relevant but simple behavioral tests which are based on motoric output and allow for automated supervision and machine learning analysis. They conclude that the neurophysiological analysis to follow should include ongoing activity of hypothalamus and the midbrain supraspinal motor system, structures hypothesized to be serving a hierarchical role that primes rather than follows (neo)cortical activity.

In their mini-review, Canonica and Zalachoras, highlight the centrality of motivational deficits in neuropsychiatric disorders, which are often overlooked in animals and clinical studies, and their dependence on dopaminergic systems.

Finally, Mallien et al. and Reiber et al., introduce the value of employing animal welfare principles based on species-specific behaviors and sex-specific correlates as a means to strengthen experimental rigor and translational potential in knock-out (KO) models of neuropsychiatric disorders. More specifically, Reiber et al. showed that Glu1A deficient mice show a pattern of peri-adolescent time-limited behavioral impairment, in terms of burrowing and nesting, which was attributed to novelty-induced hyperactivity, while adrenocortical activity in pre-puberty was only evident in female Glu1A KO mice. In a similar study by Mallien et al., which places examination of wellbeing in transgenic rat models this time in the spotlight of assessing translatability of animal models, dopamine transporter (DAT) KO rats show alterations in natural behavioral patterns, like reduced borrowing and social interaction and increased stereotypies, indicative of coping impairments.

As collectively suggested by the papers in this selection, including ethologically relevant aspects and principles of animal welfare in the experimental design of behavioral approaches, as well as taking into account age and sex as modifiers of behavioral manifestations of disease aspects will benefit face validity and translatability of human behavioral disorders greatly. When modeling mental health disorders in animals the use of the following strategies will aid greatly in designing informative neurophysiological experiments: (i) semi-natural settings and (ii) continuous long-lasting recording of full behavioral repertoires, followed by (iii) supervised machine learning algorithms for identifying nuanced behavioral sequences, beyond overused simplistic behavioral outputs (Shemesh and Chen, 2023). In order to further increase translational validity, an increased effort needs to be placed on the integration of domain and symptom specific models of human mental health disorders that are causally linked to neuronal pathways or neurobiological changes (Schmack et al., 2022). As no single animal model or task can encompass the full complexity of a given psychiatric disorder (Becker et al., 2021), pluralism in animal modeling and testing for covering and collectively mimicking multiple aspects of the human disease is a necessary element for preclinical neuropsychiatric research, while crosstalk between behavioral science and philosophy has been suggested to benefit such endeavors further (Krakauer et al., 2017; Laplane et al., 2019). Lastly, adopting clear conceptual and terminology definitions of psychological, cognitive and emotional concepts would help systematize and deconstruct the different distinct aspects of behavioral protocols, “unlocking” our access to potentially different underlying brain circuits and neurotransmitter/neuromodulator systems and improving the correspondence between discrete behavioral readouts and brain functions.

## Author contributions

ST and AD wrote the article in consultation with JP and AS. JP and AS edited the final version of the manuscript. All authors contributed to the article and approved the submitted version.
